# Not racemic after all: stereochemical composition of a commercially available sodium lactate solution

**DOI:** 10.1186/s13054-026-05992-0

**Published:** 2026-04-02

**Authors:** Mette Glavind Bülow Pedersen, Robert Andrew Robergs, Kristoffer Berg-Hansen, Esben Søndergaard, Niels Møller, Nikolaj Rittig

**Affiliations:** 1 Steno Diabetes Center Aarhus, Aarhus University Hospital, Aarhus N, Denmark; 2 University of Jan Evangelista Purkyne (UJEP), Ústí nad Labem, Czech Republic; 3 Department of Anesthesiology and Intensive Care, Horsens Regional Hospital, Horsens, Denmark; 4 Department of Cardiology, Aarhus University Hospital, Aarhus N, Denmark; 5 Department of Endocrinology and Internal Medicine, Aarhus University Hospital, Aarhus N, Denmark; 6 Department of Internal Medicine, Horsens Regional Hospital, Horsens, Denmark; 7 Medical/Steno Aarhus research laboratory, Aarhus University, Palle Juul- Jensens Blvd 11, 8200 Aarhus N, Denmark

## To the Editor

Lactate infusions have been widely employed for decades across experimental human physiology and clinical studies. Although often treated as a single entity, lactate exists as two enantiomers: L-lactate, the dominant physiological form derived from glycolysis, and D-lactate. In humans, D-lactate is generated in vivo through methylglyoxal detoxification and circulates at systemic concentrations that represent only a very small fraction of the L-lactate enantiomer [1]. Hypertonic sodium lactate (HSL) therapy has shown potential in neurology and cardiology, yet most clinical studies do not disclose the lactate enantiomer composition of exogenous lactate therapies [1]. In a recent publication in *Critical Care*, our group reported beneficial cardiovascular effects of a commercially available HSL compound [2]. This HSL compound is produced by Monico SpA (Italy) and is - according to the Summary of Product Characteristics and confirmation from the importer (Specific Pharma A/S, 2021) - a racemic formulation. A racemic compound is generally defined as containing an equal amount of two enantiomers [3]. For lactate, this implies equal proportions of L- and D-lactate. We and others have used this compound in clinical and preclinical studies [2, 4–9], (NCT06121323, NCT06265337). Others are currently using HSL by Monico SpA in a phase II trial involving comatose patients after cardiac arrest (NCT05004610).

Our use of a racemic lactate formulation has prompted scientific debate in *Critical Care* [10–13] primarily due to concerns about potential deleterious effects of D-lactate accumulation in critically ill patients. In contrast, studies in healthy individuals report no harmful effects of D/L-lactate administration, even at plasma D-lactate concentrations up to 5 mmol/L [1]. Thus, the physiological and clinical relevance of the D-lactate enantiomer remains an important knowledge gap in the context of lactate therapy. Nevertheless, we have acknowledged the unphysiological enantiomer distribution as a limitation of prior work [7].

**Fig. 1:**
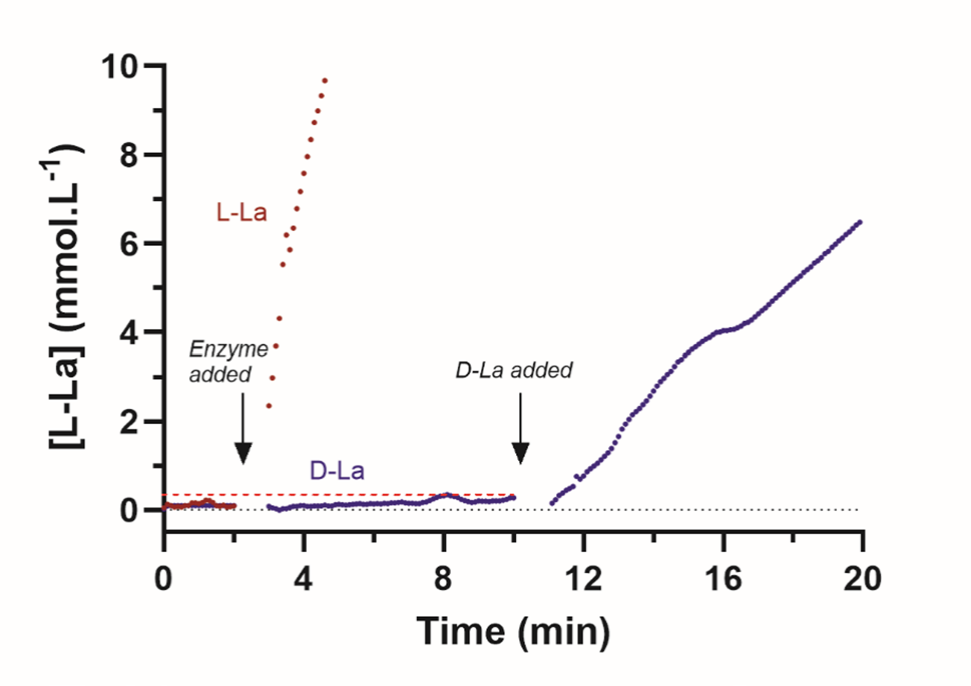
Sample data from the initial kinetic spectrophotometric assays of L- and D-lactate samples from a diluted sample of a commercial 2 M lactate solution. The data from two different assays (L-Lactate vs. D-Lactate) are overlayed and adjusted to similar baseline values to improve comparisons. Note the gradual small increase in the D-Lactate concentration (-------) once the enzyme was added. The further increase in D-Lactate concentration after pure D-Lactate was added after 10 min confirms the viability of the reagent and adequate activity of the D-lactate dehydrogenase enzyme to detect D-Lactate. L-La: L-Lactate. D-La: D-Lactate

Prompted by this debate, we recently sought to verify the enantiomeric composition of our HSL compound. Because standard lactate measuring devices (e.g.., blood gas analyzers and YSI analyzers) quantify only L-lactate, they do not provide information on the enantiomeric composition. We therefore collaborated with Prof. Robert Robergs (Czech Republic), who performed a series of enzymatic assays using D- and L-lactate dehydrogenase isoforms with spectrophotometric quantification of NADH. The assay approach underwent several independent rounds of refinement, incorporating multiple dilution schemes, enzyme-specific kinetic conditions, and both room-temperature and heated acid-hydrolysis steps prior to the reagent driven assay to ensure full recovery of detectable lactate. Parallel measurements using known L- and D-lactate standards were performed to confirm assay specificity and linearity. Across all assay rounds, results consistently demonstrated a high L-lactate concentration and a D-lactate fraction of < 1%, rather than the nominal 50% assumed for the compound. Figure 1 provides an example of the initial spectrophotometric kinetic analyses of the sample that reveals minor D-Lactate presence in the commercial solution compared to the relative abundance of L-Lactate. The analyses also indicated a small proportion of short-chain lactide species [14], inferred from a proportion of lactate content recoverable only after acid hydrolysis. While complete datasets and methodological details will be reported in a full manuscript currently in preparation, we believe that the emerging pattern of findings warrants early communication due to its potential clinical implications.

With respect to our previous studies, this new information does not change the findings but simplifies their interpretation: the physiological effects we observed are most likely attributable to L-lactate. Although speculative, the very low D-lactate content in our solution may also explain why we observed much milder side effects following oral ingestion of the compound [6] compared with another group that used a 60% racemic sodium lactate solution and reported severe side effects, including diarrhea and vomiting [15].

In the interest of scientific transparency and reproducibility, we recommend that future investigations explicitly verify and report the stereochemical profile of lactate preparations. As the field increasingly moves from physiological studies towards therapeutic application of lactate, clarifying this aspect becomes essential for accurate interpretation of experimental findings, comparability across studies, and guiding the effective clinical use of lactate-based interventions.

## Data Availability

The data that support the findings of this study are available from the corresponding author upon reasonable request.
